# Deep-learning magnetic resonance imaging-based automatic segmentation for organs-at-risk in the brain: Accuracy and impact on dose distribution

**DOI:** 10.1016/j.phro.2023.100454

**Published:** 2023-06-06

**Authors:** Andrada Turcas, Daniel Leucuta, Cristina Balan, Enrico Clementel, Cristina Gheara, Alex Kacso, Sarah M. Kelly, Delia Tanasa, Dana Cernea, Patriciu Achimas-Cadariu

**Affiliations:** a The European Organisation for Research and Treatment of Cancer (EORTC) Headquarters, RTQA, Brussels, Belgium; b SIOP Europe, The European Society for Paediatric Oncology (SIOPE), QUARTET Project, Brussels, Belgium; c University of Medicine and Pharmacy and Medicine “Iuliu Hatieganu”, Oncology Department, Cluj-Napoca, Romania; d Oncology Institute “Prof. Dr. Ion Chiricuta”, Radiotherapy Department, Cluj-Napoca, Romania; e University of Medicine and Pharmacy “Iuliu Hatieganu”, Department of Medical Informatics and Biostatistics, Cluj-Napoca, Romania; f “Babes-Bolyai” University, Faculty of Physics, Cluj-Napoca, Romania; g Oncology Institute “Prof. Dr. Ion Chiricuta”, Surgery Department, Cluj-Napoca, Romania

**Keywords:** Radiotherapy, Artificial Intelligence, Radiation Oncology, Brain tumors, Delineation/contouring, Treatment planning

## Abstract

**Background and purpose:**

Normal tissue sparing in radiotherapy relies on proper delineation. While manual contouring is time consuming and subject to inter-observer variability, auto-contouring could optimize workflows and harmonize practice. We assessed the accuracy of a commercial, deep-learning, MRI-based tool for brain organs-at-risk delineation.

**Materials and methods:**

Thirty adult brain tumor patients were retrospectively manually recontoured. Two additional structure sets were obtained: AI (artificial intelligence) and AIedit (manually corrected auto-contours). For 15 selected cases, identical plans were optimized for each structure set. We used Dice Similarity Coefficient (DSC) and mean surface-distance (MSD) for geometric comparison and gamma analysis and dose-volume-histogram comparison for dose metrics evaluation. Wilcoxon signed-ranks test was used for paired data, Spearman coefficient(ρ) for correlations and Bland–Altman plots to assess level of agreement.

**Results:**

Auto-contouring was significantly faster than manual (1.1/20 min, p < 0.01). Median DSC and MSD were 0.7/0.9 mm for AI and 0.8/0.5 mm for AIedit. DSC was significantly correlated with structure size (ρ = 0.76, p < 0.01), with higher DSC for large structures. Median gamma pass rate was 74% (71–81%) for Plan_AI and 82% (75–86%) for Plan_AIedit, with no correlation with DSC or MSD. Differences between Dmean_AI and Dmean_Ref were ≤ 0.2 Gy (p < 0.05). The dose difference was moderately correlated with DSC. Bland Altman plot showed minimal discrepancy (0.1/0) between AI and reference Dmean/Dmax.

**Conclusions:**

The AI-model showed good accuracy for large structures, but developments are required for smaller ones. Auto-segmentation was significantly faster, with minor differences in dose distribution caused by geometric variations.

## Highlights

• Deep-learning-based automatic segmentation of brain organs-at-risk was fast and accurate.
• Automatic contouring reduced inter-observer variability.
• Dice similarity coefficients correlated with size, with values > 0.8 for larger structures.
• Geometric variations had minimum impact on dose distribution.

## 1 Introduction

Radiotherapy is part of the multidisciplinary management of both primary and metastatic brain tumours, frequently in combination with systemic therapy and surgery. Each of these treatment modalities carry individual risk of adverse effects, which is increased when they are combined [1], [2], [3], [4], [5]. Radiation-induced acute toxicities can be dose-limiting or life threatening, and late effects such as hearing or visual impairment, endocrine disfunction, and neuro-cognitive decline can be detrimental for long-term survival patients [6], [7], [8].

Delineation of organs-at-risk (OARs) is essential for radiotherapy planning, enabling healthy tissue sparing and toxicity risk estimation. With the availability of high-quality imaging such as magnetic resonance imaging (MRI), an increasing number of brain structures can be accurately delineated; including them in the plan optimization process can help to minimize the risk of adverse events, increase the therapeutic ratio, and improve the quality of survival. However, manual contouring is often challenging in terms of time burden and accuracy – considerable variability still exists between observers [9], [10], [11] despite the availability of anatomical guidelines and atlases [12], [13]. Variations in contours can impact dosimetry and plan quality, with subsequent potential impact on treatment outcomes [14], [15], [16]. Several automatic and semi-automatic tools for target and OAR delineation are currently available or under investigation, including traditional atlas-based or machine-learning approaches and innovative artificial intelligence (AI) neural networks/deep learning-based algorithms. These methods could potentially optimize workflows, shorten planning times, and harmonize practice by reducing inter- and intra-observer variability in delineation [17], [18]. Some AI-models have shown good results in terms of contouring accuracy, with several programs already commercially available [14], [19], [20]. However, it is not yet established if a faster delineation translates into a faster overall planning time, or if the existing geometric discrepancies in contouring have any impact on the dose distribution when compared to the current human-based standard.

In this study we evaluated the feasibility and performance of using a T1-MRI model of a commercially available, guideline-based, deep-learning automatic segmentation software for organ-at-risk delineation in the brain. We addressed the workflow, time burden, and geometric accuracy of the AI-assisted method compared to the human/clinician-based process and investigated the potential impact of the geometric differences on dose distribution, to clinically validate this method on an independent data set.

## 2 Material and methods

This retrospective study was reviewed and approved by the local ethics committee. All patients have previously consented for their data to be used for research purposes and all patient data was anonymized prior to this investigation.

## 3 Patients, delineation and geometric comparison

Thirty adult (>18 years) patients with primary brain tumors that had previously completed radiotherapy in a tertiary cancer center were randomly selected. A set of 22 OARs per patient were manually contoured by a radiation oncologist specialized in brain radiotherapy, based on the EPTN (European Particle Therapy Network) recommendations [12], [13], using the planning computed tomography (CT) rigidly registered with a gadolinium enhanced T1-weighted MRI scan. The patients had tumours in various locations: occipital (n = 8), parietal (n = 8), temporal (n = 7), midline (n = 4), frontal (n = 2) and the posterior fossa (n = 1). Most of the hemispheric tumours were on the left (16) and nine were on the right side. Four scans were acquired with 3Tesla MRI scanners and 26 with 1.5Tesla machines. Slice thickness was one millimeter or less for 17 MRI scans and 1.5–5 mm for the other 13. Slice thickness was proportional with the total number of slices, the latter being further used as a surrogate for the analysis. Manual delineations were expert approved and considered as the reference/ground-truth structures. For each case, a second segmentation was then performed using a commercial, deep-learning, guideline-based artificial intelligence (AI) software (MVision GBS™, Version 1.2.2). The T1-MRI Brain model v1.2.2 consists of a convolutional neural network with encoder-decoder architecture, which was trained using an ADAM optimizer on 90 cerebral 3D-iso T1-MRI (1.5Tesla), following the EPTN contouring guidelines [21]. The AI structures were then uploaded into the local planning software and transferred to the rigidly registered planning CT. Subsequently, AI structures were manually edited, until they were considered clinically acceptable, thus obtaining a third structure set (AIedit). Delineation time for each step was documented.

To capture inter-observer variability, five cases were randomly selected for a contouring exercise in which two radiation oncology trainees (RO A and B) manually delineated the 22 OARs, thus having five alternative structure sets for these cases (RO_A, RO_B, AI, AIedit, and Reference).

For geometric comparison, Velocity™ software (Varian Medical Systems, Palo Alto, CA, USA) was used to compute the Dice Similarity Coefficient (DSC) [22] and surface distances (mean = MSD, median, maximum = Hausdorff Distance, HD). The “union” function was used to merge all structures except the Brain (which includes all organs but the Brainstem and its’ subdivisions), obtaining one single structure encompassing 21 OARs (Union_Ref, Union_AI, Union_AIedit, Union_RO-A, Union_RO-B).

### 3.1 Planning and plan comparison

Volumetric-modulated arc therapy plans were generated for 15 selected representative cases using the same plan objectives and optimization criteria as the original plan, defined within the institutional protocol, which is based upon the EPTN consensus recommendations [23]. A dose of 60 Gy (standard fractionation) was prescribed to the Planning Target Volume (PTV) [24]. Each plan was re-optimized based on the three structure sets, (Plan_Ref, Plan_AI, Plan_AIedit) thus obtaining 45 different dose distributions. PTV and any originally contoured OARs not included in the 22 test structures (Cochlea_L/R, Eye_L/R, Lens_L/R, Retina_L/R) were copied from the original plans (previously used for patient treatment). Both the manual delineations and treatment planning were performed using Eclipse 15.6 (Varian Medical Systems, Palo Alto, USA). Dose-Volume-Histogram (DVH) comparison and Dmean/Dmax collection for each plan was performed based on the reference structures, as they were considered to best reflect the real anatomy, using the DICOM registration and plan comparison functions in Velocity. Dose differences were defined as follows: ΔDmean_AI/AIedit = Dmean_Ref minus Dmean_AI/AIedit and ΔDmax_AI/AIedit = Dmax_Ref minus Dmax_AI/AIedit, in absolute values. SNC Patient 8.2.0 (Sun Nuclear Corporation, Florida, USA) was used for relative gamma analysis with 3%/3mm pass criteria and a 10% dose cut-off.

### 3.2 Statistical analysis

Wilcoxon signed-ranks test and Bland Altman plots were used for paired, non-normal distributed data, correlations were assessed with the Spearman correlation coefficient(ρ), and volume thresholds were calculated with receiver operating characteristic (ROC) curves, using the R software (R Foundation for Statistical Computing, Vienna, Austria). A detailed description of the workflow is shown in Supplementary material-Figure S1.

## 4 Results

### 4.1 Workflow

With a median time of 1.1 min (Interquartile Range (IQR) 0.9–2.4 min) for AI generation and 20 min (IQR 18–23.5 min) for manual delineation, the automatic tool was significantly faster (p < 0.05). There was a positive correlation between the segmentation time and the number of MRI slices for AI (ρ = 0.8, 95 %CI 0.3–0.8, p < 0.05), but not for manual contouring (ρ = 0.05, 95 %CI −0.34–0.33, p = 0.79). Also, the AI-contouring time was positively correlated with the DSC (ρ = 0.5, 95 %CI 0.2–0.7, p < 0.05). Reported time for editing the AI-generated structures was 15 min, compared against an average delineation time for RO_A and RO_B of 40–60 min.

**Fig. 1:**
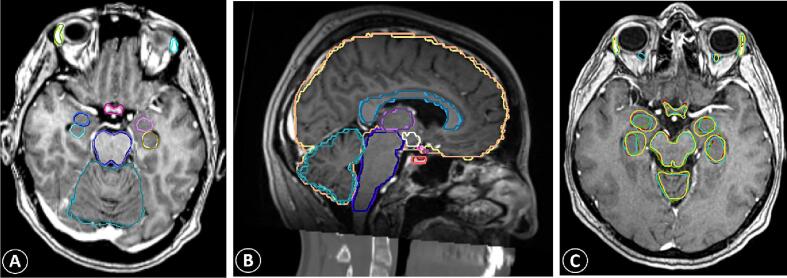
**A, B-** Example case with reference structures in darker and AI-generated structures in lighter shades. **C:** Axial MRI showing inter-observer variability between clinicians (yellow and green), reference structures (cyan) and AI contours (red).

The automatic tool failed to generate 27 of the 660 expected OARs (4%), three of which missing because of tumour invasion, thus having an overall success rate of 96% for delineated structures. Eleven cases had at least one missing structure, with an average of 0.9 structures per case. The OpticChiasm was missing in six patients, followed by the CorpusCallosum (n = 4), OpticTract_L/R (n = 2), and Pituitary (n = 2). Other missing structures were OpticNrv_L/R and GlndLacrimal_L/R (n = 2/1 and 1/2). Fig. 1**: A, B-** shows an example of a representative case.

### 4.2 Geometric analysis

Median DSC between AI and Reference for all 22 structures was 0.73 (IQR0.55–0.82). DSC (for each individual structure) was strongly correlated with the structure volume (ρ = 0.76, 95% Confidence Interval (CI) = 0.72–0.80, p < 0.05), with DSC ≥ 0.80 for structures>4.05 cm^3^ (Specificity = 0.89, Sensibility = 0.78; Area Under Curve = 0.86, 95 %CI 0.83–0.9). The highest DSC was calculated for Brain (0.96), Cerebellum (0.92), Brainstem (0.89), whereas the lowest was found for the smaller structures: GlndLacrimal_L/R (0.45/0.55) and OpticTract_L/R (0.45/0.48). Median DSC between Union_Ref and Union_AI (DSC_AI) was 0.89 (IQR0.87–0.91) and was positively correlated with the number of MRI slices (ρ = 0.65, 95 %CI 0.18–0.81, p < 0.05).

Median MSD was 0.9 mm (IQR0.6–1.4 mm), lowest for Hippocampus_R,Amygdala_L and OpticChiasm (0.6 mm), and largest for MedullaOblongata (1.7 mm), Pons (1.5 mm) and OpticNrv_L (1.4 mm). Median surface distance was zero mm for four OARs (Hippocampus_R/L, OpticChiasm and OpticNrv_R), with MedullaOblongata and Midbrain having the highest median values (1.3 mm, 1.3 mm). HD was lowest for Amygdala_L/R (3.6/3.7 mm) and highest for Brain (20.9 mm). The complete geometric comparison data is presented in the Supplementary material- Table S1.

Median DSC between AIedit and Reference structures was 0.82 (IQR 0.69–0.89), with highest DSC for the larger OARs: Brain (0.96), Cerebellum (0.92), Brainstem (0.90) and Pons (0.87), and lowest for OpticTract_L/R (0.64/0.60) and GlndLacrimal_L/R (0.64/0.64). Overall DSC_AIedit was 0.90 (IQR0.89–0.92). Median MSD was 0.5 mm (IQR0.2–1), lowest for Hippocampus_L/R (0.1 mm) and Amygdala_L/R (0.2 mm) and highest for Pons (1.2 mm), MedullaOblongata (1.1 mm) and Midbrain (1.0 mm). Median surface distance was zero for 13 OARs, and the highest value was observed for Pons (0.5 mm). The lowest HD was 1.9/2.0 mm for Amygdala_R/L and highest for Brain (11.5 mm).

**Fig. 2:**
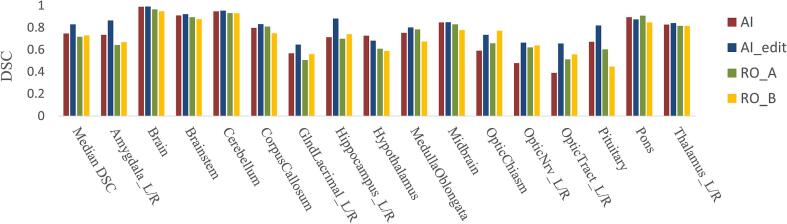
Dice Similarity Coefficient (DSC) between auto-segmented (AI), manually manipulated auto-segmented (AIedit), and clinician-based (RO_A, RO_B) contours against the reference structures. Median DSC = median value of all calculated DSCs; For paired OARs, we report the median between left and right.

For the five selected cases that were additionally contoured by two clinicians, median DSC for all OARs was 0.71 for RO_A (IQR 0.59–0.82) and 0.72 for RO_B (IQR 0.58–0.81), whereas median DSC was 0.74 (IQR 0.58–0.84) for AI and 0.82 (IQR 0.69–0.88) for AIedit. DSC between Union_Ref and Union_RO_A/B was 0.89/0.88. Lowest median DSC for RO_A was 0.44 for GlndLacrimal_L and 0.47/0.54 for OpticTract_R/L and for RO_B was for GlndLacrimal_R/L (0.55/0.56), Pituitary (0.44) and OpticTract_R/L (0.54/0.56). Highest median DSC for both RO_A and RO_B were for Brain (0.96/0.94), Cerebellum (0.93/0.92) and Brainstem (0.89/0.87). Inter-rater intra-class correlation coefficient was 0.66 (95 %CI 0.54–0.75, p < 0.05), indicating a moderate agreement between the two raters. Fig. 1**C** and Fig. 2 represent visual and graphical comparisons of the five structure sets for the selected patients.

### 4.3 Dose-volume-histogram and gamma analysis

**Fig. 3:**
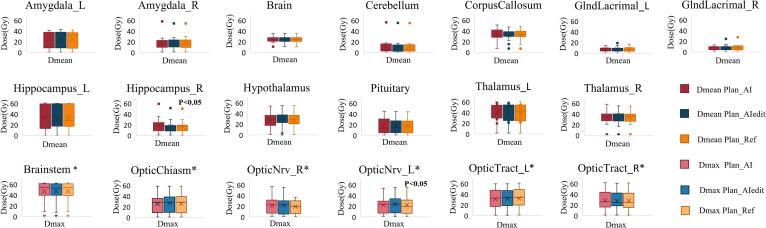
Box plots showing the difference in mean doses (Dmean) and maximum doses (Dmax, marked with*, for serial OARs only). Where differences were statistically significant, p < 0.05 was added to the plot.

**Fig. 4:**
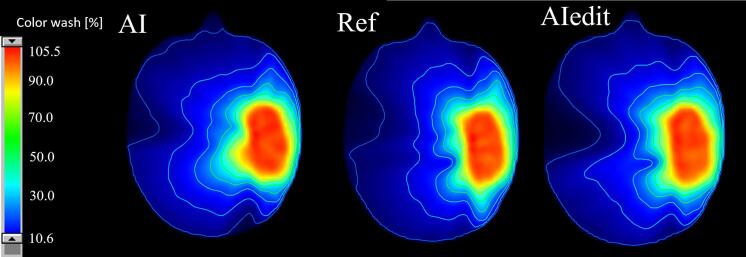
Dose distributions for one representative case, based on AI, Reference and AIedit contours.

Dmean and Dmax largely varied across OARs, depending on tumour location (Fig. 3). The median gamma pass rate (3%/3mm, 10% threshold) was 74% (IQR 71–80.7%) between Plan_Ref and Plan_AI and 82% (IQR 75–86%) between Plan_Ref and Plan_AIedit (Fig. 4, Supplementary material Figure S2, S3), without any correlation with DSC or MSD.

**Fig. 5:**
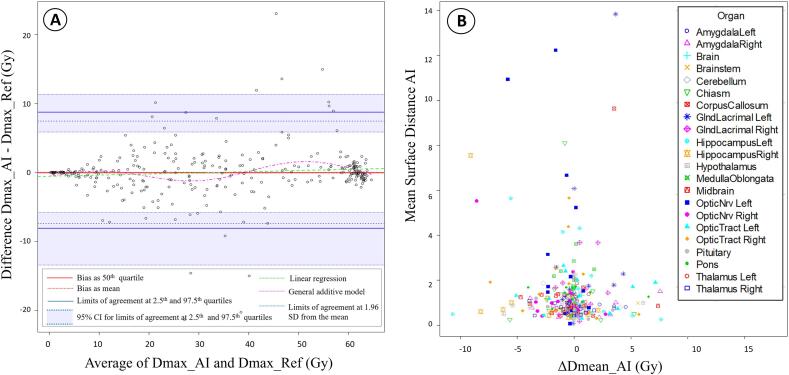
**A**: Bland-Altman plot showing the level of agreement between Plan_AI and Plan_Ref, in terms of Dmean; **B**: Scatter plot showing the correlation between ΔDmean_AI (Gy) with DSC_AI for each OAR.

Overall, there were small differences (<0.2 Gy) between median values for Dmean_AI/Dmean_Ref and Dmax_AI/Dmax_Ref, respectively. The Bland-Altman plots in Fig. 5**A** and Supplementary material- Fig. S4a demonstrate the difference between Dmean/max_AI and Dmean/max_Ref being minor, with an average discrepancy (bias) of 0.1 (95 %CI 0–0.2) for Dmean and zero (95 %CI −0.1–0) for Dmax, along with narrow limits of agreement. Dmean_AI was significantly higher (p < 0.05) than Dmean_Ref for Hippocampus_R, Midbrain, and OpticNrv_L/R and lower for MedullaOblongata, whereas Dmax_AI was higher than Dmax_Ref for Hippocampus_R and Thalamus_L, and lower for Amygdala_L (p < 0.05). No difference was found for other OARs.

The differences between median values for Dmean_AIedit/Dmean_Ref and Dmax_AIedit/Dmax_Ref were smaller than 0.5 Gy. Individually, statistical significance(p < 0.05) was only reached for OpticChiasm and OpticNrv_L, with higher Dmean for AIedit. Dmax_AIedit was higher for Hypothalamus and OpticNrv_L and lower for GlndLacrimal_R and Pons (p < 0.05).

Highest and lowest absolute (Gy) and relative (%) dose differences (ΔD) are shown in Supplementary material - Table2. ΔDmean_AI(Gy) was negatively correlated with DSC_AI (ρ = -0.26, 95 %CI-0.36- −0.13, p < 0.05) (Fig. 5B), but significant correlation (ρ = 0.56, p < 0.05) was only confirmed for Hippocampus_R in the individual OAR analysis. DSC_AI was also correlated with absolute and relative ΔDmax_AI (ρ = -0.21, 95 %CI-0.29- −0.01/ρ = -0.26, 95 %CI-0.33– −0.07, p < 0.05), with individual significance for Hippocampus_R (ΔDmax_AI%) and Brainstem (ΔDmax_AI Gy). MSD_AI was not significantly (p = 0.3) correlated with absolute or relative ΔDmean_AI (Supplementary material - Fig. S4b) or ΔDmax_AI, but a slight correlation was found for Hippocampus_R (ρ = 0.56 for ΔDmean/ρ = 0.67 for ΔDmax_AI, p < 0.05).

No statistically significant difference was found between Dmean/max_AI and Dmean/max_AIedit.

## 5 Discussion

In this study, we evaluated an automatic segmentation tool for brain OARs in a clinical setting, with focus on workflow, time, and accuracy for reducing inter-observer variability. Furthermore, we tested the feasibility of using the AI-generated contours for planning, with or without subsequent human intervention and manual adjustment. The clinical validation of this deep learning-based AI tool was performed on an independent data set than the one used for training the model. The automatic segmentation took significantly less time than manual contouring. However, additional time was needed for other manually performed steps: exporting and importing structures in the local planning system, transferring structures on the planning CT and potential corrections.

One of the strengths of this study is the large number of OARs (22) included and the use of MRI for delineation, which is essential in brain radiotherapy. This is valuable especially in the era of intensity-modulation and inverse planning, where inclusion of OAR in the optimisation process is essential to improve OAR sparing. Other authors have mostly reported on a smaller number of brain OARs, with focus on optic nerves, chiasm, brainstem, and hippocampi (Supplementary material - Figure S5) [15], [17], [19], [25], [26], [27], [28], [29], [30]. There is limited data reporting on MRI-based auto-segmentation in the brain, as most of the reports are CT-oriented [16], [18], [31], [32], [33], [34], [35], [36], [37]. Nonetheless, some OARs (Cochlea, Eyes, Retina, and Lenses) are not included in this model and are of high importance for radiotherapy planning and would require manual delineation. These, together with GlndLacrimal_L/R, OpticNrv_L/R, are generally better visualised on CT, which might explain the lower DSC when contoured on the MRI scan, with several reports showing better results for CT-based segmentation [33], [37], [38].

Our results indicate that auto-segmentation can reduce inter-observer variability, with similar or higher DSC reported for AI-generated contours for most OARs, when compared to RO_A and RO_B. The overall DSC was very good for the AI-generated structures and increased after the manual editing, leading to smaller differences in dose distribution, but adding a considerable amount of time. Poorer results were found for small structures, especially the optic apparatus, with DSC values around 0.45–0.55, however these structures are usually rapidly delineated by clinicians. Nonetheless, DSC has its limits in evaluating contour similarity, as it is sensitive for small structures, but it becomes less sensitive for larger ones. Other used metrics, such as the surface distances, were highest for the large structures (Brain, Brainstem, and its subdivisions), mostly caused by discrepancies in superior and inferior borders of these structures. There are alternative geometric parameters available which may bring additional information on the accuracy of the auto-segmentation tool, metrics such as surface dice coefficients and Jaccard index, [39] which we did not use in this study. The exclusion of these metrics could be considered a limitation, along with the subjective matter of assigning a ground truth, as no delineation, despite expert-approval, can perfectly replicate real anatomy and is performed on a single snap-shot time point that does not always represent the anatomy. It should be noted that, distortions of the contours might have occurred during the MRI-to-CT transfer, likely due to imperfect registration, variations in CT/MRI slice thickness and different scanning angles. The correlation between the DSC and the number of MRI slices (which was used as a surrogate for slice thickness) indicates that for optimal results a good quality MRI scan is needed.

Our results showed that plans optimized on raw, AI-based contours were similar to those based on the reference contours, with only minor differences. Dmean values were higher for Plan_AI and conversely, Dmax was slightly lower for Plan_AI, suggesting that some differences might be random or caused by other factors than delineation. One such factor could be the optimization process, which might deliver slightly different solutions to the same problem, even if very similar structures and identical criteria are used for planning. Therefore, we believe that reported differences in dose metrics are not exact reflections of variations in geometry and statistical differences do not necessarily translate into clinical significance. Still, ΔDmean and ΔDmax showed a modest negative correlation with DSC, meaning that lower DSC might lead to a bigger dose difference, but was not confirmed on an individual OAR basis. These results are supported by the Bland Altman test, which showed minimal/no bias and narrow limits, suggesting that the two methods are nearly identical. The gamma pass rate showed a good similarity between dose distributions for most plans, with some lower values, mostly in cases with missing structures. Other authors also noted that contouring variations might impact dose distribution, but these results should be carefully interpreted in terms of clinical relevance, given the multi-factorial aspects of dose calculation and distribution [14], [15], [16], [40].

Considering all aspects, deep-learning, MRI-based auto-segmentation for brain OARs can be a valuable tool in clinical practice, with good accuracy especially for large structures, but further developments are required for smaller organs. This method could significantly reduce work burden, shorten treatment preparation time, and reduce inter- and intra-observer variability, with a clinically acceptable impact on the dose distribution.

## Declaration of Competing Interest

The authors declare that they have no known competing financial interests or personal relationships that could have appeared to influence the work reported in this paper.
