# Supplementary material for: Deep-learning magnetic resonance imaging-based automatic segmentation for organs-at-risk in the brain: Accuracy and impact on dose distribution
Source: Phys Imaging Radiat Oncol. 2023 Jun 6;27:100454. doi: 10.1016/j.phro.2023.100454 (PMC10276287; doi:10.1016/j.phro.2023.100454)
Supplement: Supplementary data 1 [file mmc1.pdf]

SUPPLEMENTARY MATERIAL

Table S1- Geometric parameters for AI and AIedit structure sets compared to the Reference structures

					Surface Distance Metrics											
	Conformality (DSC)				Mean Distance (mm)				Median Distance (mm)				Maximum (Hausdorff Distance) (mm)			
	AI	IQR	AIedit	IQR	AI	IQR	AIedit	IQR	AI	IQR	AIedit	IQR	AI	IQR	AIedit	IQR
**Amygdala_L**	0.737	0.659-0.767	0.865	0.829-0.893	**0.69**	0.55-0.84	0.21	0.17-0.38	0.49	0-0.53	0.00	0-0	**3.68**	3.21-4.5	2.06	1.69-2.68
**Amygdala_R**	0.722	0.953-0.98	0.869	0.842-0.892	0.77	0.51-0.89	0.20	0.14-0.30	0.49	0-0.53	0.00	0-0	**3.76**	3.41-5	1.95	1.68-2.82
**Brain**	**0.96**	0.884-0.908	0.966	0.957-0.986	1.31	0.81-1.48	0.95	0.52-1.25	0.57	0.01-1.06	0.52	0-0.89	20.95	15.12-30.37	11.52	9.32-17.05
**Brainstem**	**0.896**	0.901-0.942	0.903	0.896-0.919	0.90	0.76-0.98	0.71	0.55-0.81	0.54	0.48-0.68	0.49	0-0.54	6.97	5.03-8.70	5.19	4.94-6.2
**Cerebellum**	**0.924**	0.726-0.823	0.926	0.915-0.944	0.88	0.67-1.32	0.87	0.64-1.02	0.49	0-0.97	0.49	0-0.55	7.50	6.51-9.97	6.56	5.81-7.65
**CorpusCallosum**	**0.792**	0.315-0.559	0.807	0.756-0.844	0.79	0.56-1.05	0.67	0.47-0.85	0.48	0-0.54	0.00	0-0.49	7.52	6.16-11.53	6.00	30838.00
**GlndLacrimal _L**	0.455	0.386-0.687	0.644	0.562-0.749	1.39	0.88-2.19	0.84	0.57-1.08	0.83	0.54-1.63	0.49	0-0.57	6.73	5.09-7.99	4.63	3.43-5.80
**GlndLacrimal _R**	0.554	0.542-0.742	0.642	0.564-0.72	1.31	0.80-2.08	0.88	0.59-1.4	0.76	0.49-1.13	0.50	0-0.69	6.85	5.02-8.06	5.08	3.80- 6.20
**Hippocampus_L**	0.66	0.61-0.769	0.871	0.846-0.891	0.75	0.49-1.50	0.15	0.11-0.19	**0.00**	0-0.54	0.00	0-0	5.52	4.19-8.29	2.50	2.01-2.62
**Hippocampus_R**	0.709	0.468-0.656	0.872	0.855-0.888	**0.67**	0.47-1.11	0.14	0.12-0.19	**0.00**	0-0.54	0.00	0-0	5.32	4.29-8.05	2.47	2.08-2.80
**Hypothalamus**	0.586	0.701-0.807	0.662	0.585-0.701	0.81	0.60-1.05	0.63	0.44-0.81	0.51	0.11-0.66	0.49	0-0.53	4.64	3.12-5.97	3.23	2.86-4.18
**MedullaOblongata**	0.751	0.791-0.841	0.813	0.772-0.844	1.73	1.22-2.21	1.19	0.85-1.59	1.07	0.74-1.54	0.53	0-0.97	7.50	5.12-9.36	5.03	4.05-5.48
**Midbrain**	**0.817**	0.337-0.67	0.837	0.802-0.881	1.25	0.87-1.35	1.07	0.41-1.21	0.92	0.51-1.09	0.49	0-0.74	5.52	5.00-6.87	4.44	3.61-6.01
**OpticChiasm**	0.546	0.278-0.603	0.733	0.657-0.8	**0.69**	0.51-1.60	0.25	0.16-0.45	**0.00**	0-1.38	0.00	0-0	4.41	3.52-6.60	2.62	2.16-3.81
**OpticNrv _L**	0.486	0.492-0.638	0.666	0.599-0.734	1.47	0.74-3.67	0.67	0.45-1.01	0.54	0-2.13	0.00	0-0	6.56	4.21-17.56	4.31	3.20-7.20
**OpticNrv _R**	0.56	0.335-0.582	0.664	0.623-0.74	0.95	0.63-1.52	0.59	0.43-0.70	**0.00**	0-0.68	0.00	0-0	5.00	3.88-11.77	4.61	3.20-5.72
**OpticTract _L**	0.456	0.388-0.551	0.644	0.549-0.741	1.26	0.76-1.64	0.76	0.33-1.03	0.62	0-1.07	0.00	0-0.51	5.88	4.57-7.68	3.87	3.12-5.19
**OpticTract _R**	0.483	0.555-0.663	0.608	0.556-0.71	0.75	0.59-1.88	0.68	0.28-1.00	0.55	0-0.93	0.00	0-0.36	4.28	3.202- 6.75	3.28	2.52-4.27
**Pituitary**	0.61	0.823-0.88	0.732	0.649-0.852	0.94	0.76-1.11	0.48	0.17-0.82	0.49	0-0.65	0.00	0-0	**3.81**	3.19-5.26	2.82	2.13-3.28
**Pons**	**0.848**	0.754-0.836	0.872	0.845-0.901	1.51	1.07-1.72	1.23	0.93-1.49	0.74	0.51-1.25	0.60	0.073-1.08	7.41	5.06-7.76	5.19	4.27-5.99
**Thalamus _L**	**0.797**	0.774-0.844	0.866	0.823-0.889	1.07	0.83-1.36	0.56	0.41-0.77	0.60	0.48-0.95	0.00	0-0.48	4.94	3.63-5.20	3.91	3.01-4.78
**Thalamus _R**	**0.815**	0.823-0.884	0.853	0.823-0.884	0.93	0.72-1.07	0.58	0.45-0.79	0.53	0.48-0.68	0.00	0-0.48	5.00	3.88-5.41	3.75	3.27-4.26

AI= Artificial Intelligence, AIedit= manually edited AI structures, DSC= Dice Similarity Coefficient, IQR= inter-quartile range;

Table S2- Highest and lowest absolute (Gy) and relative (%) dose differences (ΔD)

	Median absolute dose difference (Gy)		Median relative dose difference (%)	
	**Highest difference**	**Lowest difference**	**Highest difference**	**Lowest difference**
**ΔDmean_AI =** Dmean_Ref-Dmean_AI	Hippocampus_R -0.9Gy	OpticChiasm 0 Gy	Hippocampus_R 14%	Brain 0.3%
**ΔDmax_AI =** Dmax_Ref-Dmax_AI	Hippocampus_R -2Gy	OpticNrv_L 0 Gy	GlndLacrimal_L 16%	Brain 0.2%
**ΔDmean_AIedit=** Dmean_Ref-Dmean_AIedit	OpticNrv_L -0.9Gy	Cerebellum 0 Gy	GlndLacrimal_L/R 9%	Brain 0.5%
**ΔDmax_AIedit=** Dmax_Ref-Dmax_AIedit	GlndLacrimal_R 0.7Gy	OpticChiasm 0Gy	GlndLacrimal_L/R 10%	CorpusCallosum 0.3%

ΔD= dose difference; Gy=Gray; Dmean= mean dose; Dmax= maximum dose;

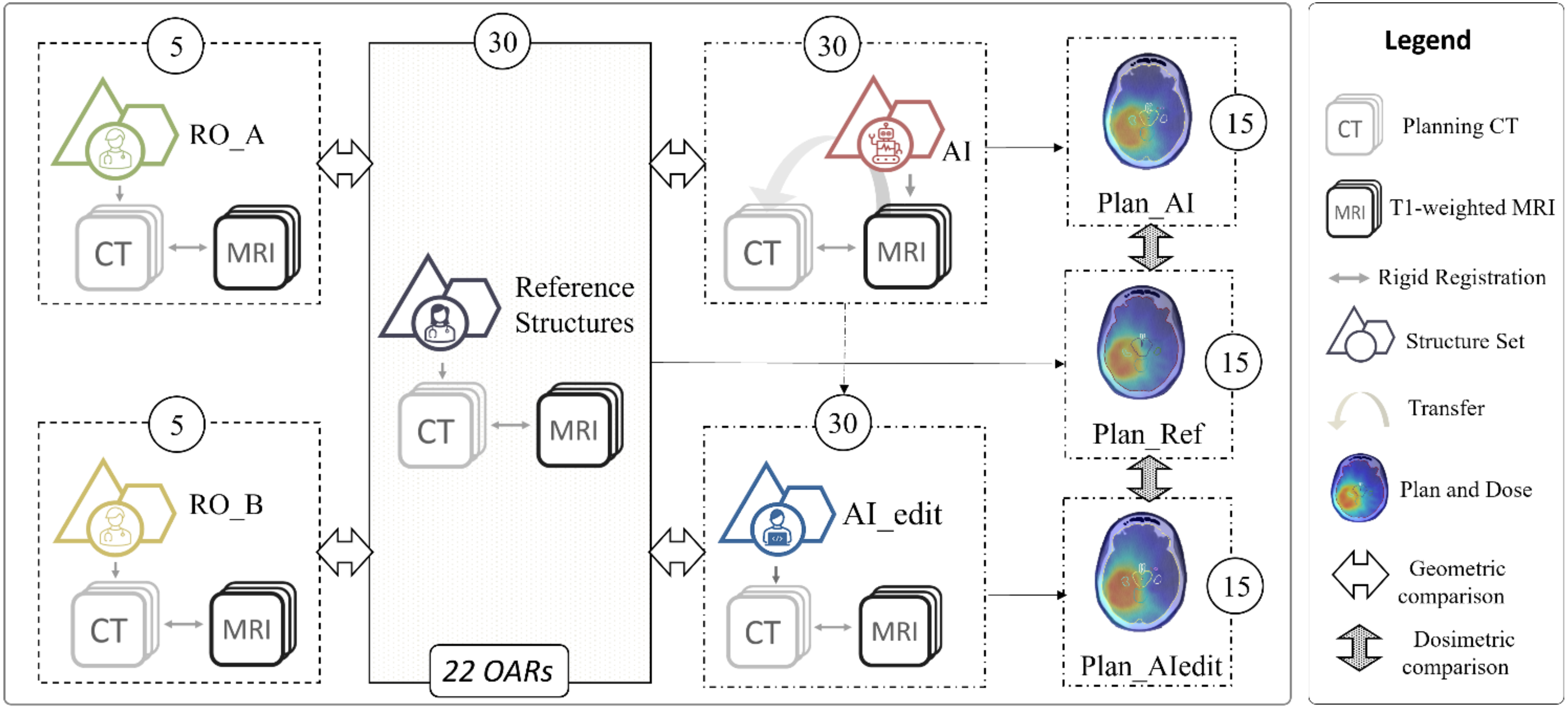

Figure S1. Study workflow. RO= Radiation oncologist, CT= computed tomography, MRI= magnetic resonance imaging, AI= artificial intelligence, Ref= reference, AIedit= manually edited AI-generated contours; OARs= organs-at-risk.

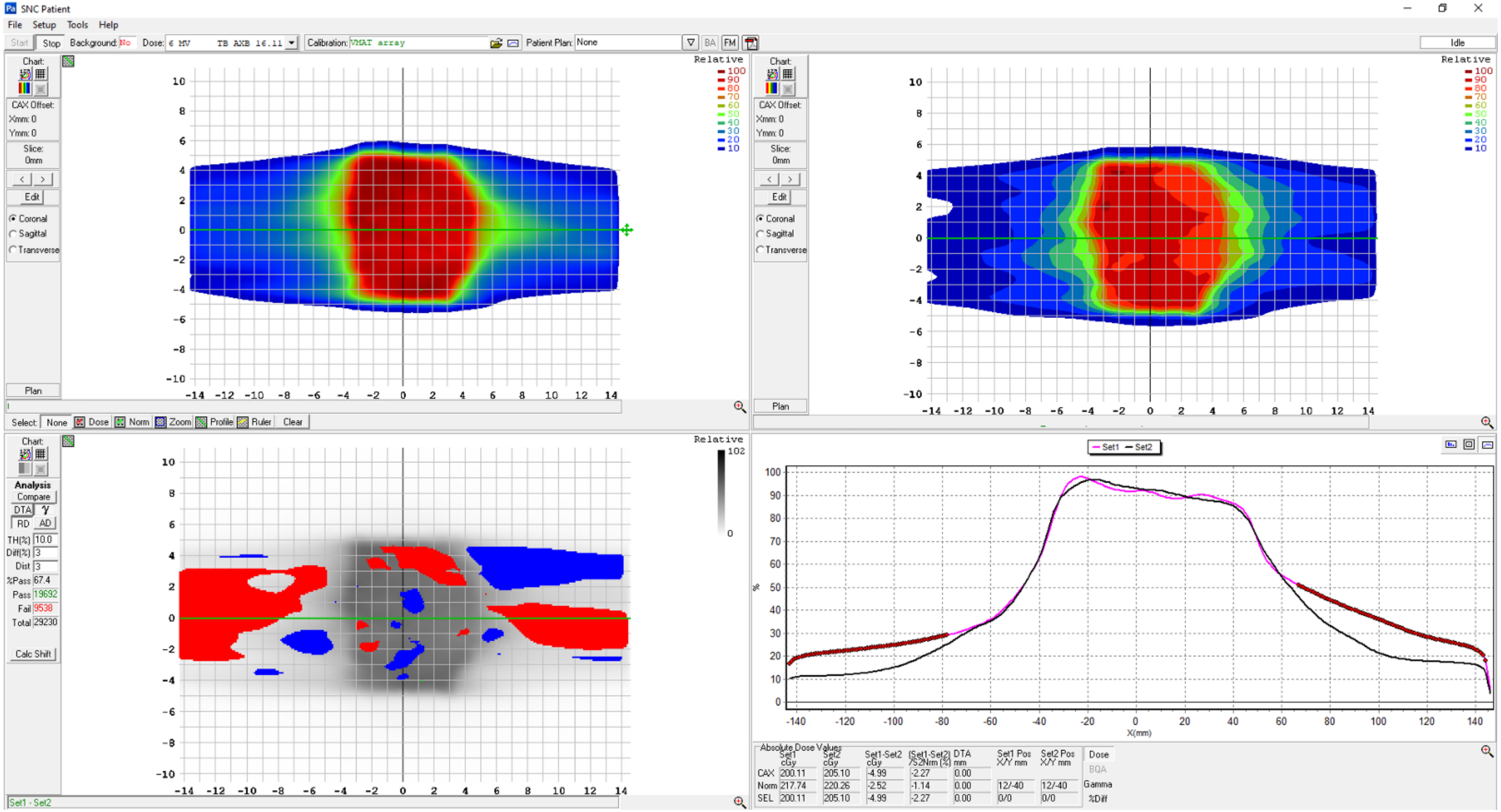

Figure S2. Gamma analysis between Plan_Ref (left) and Plan_AI (right)

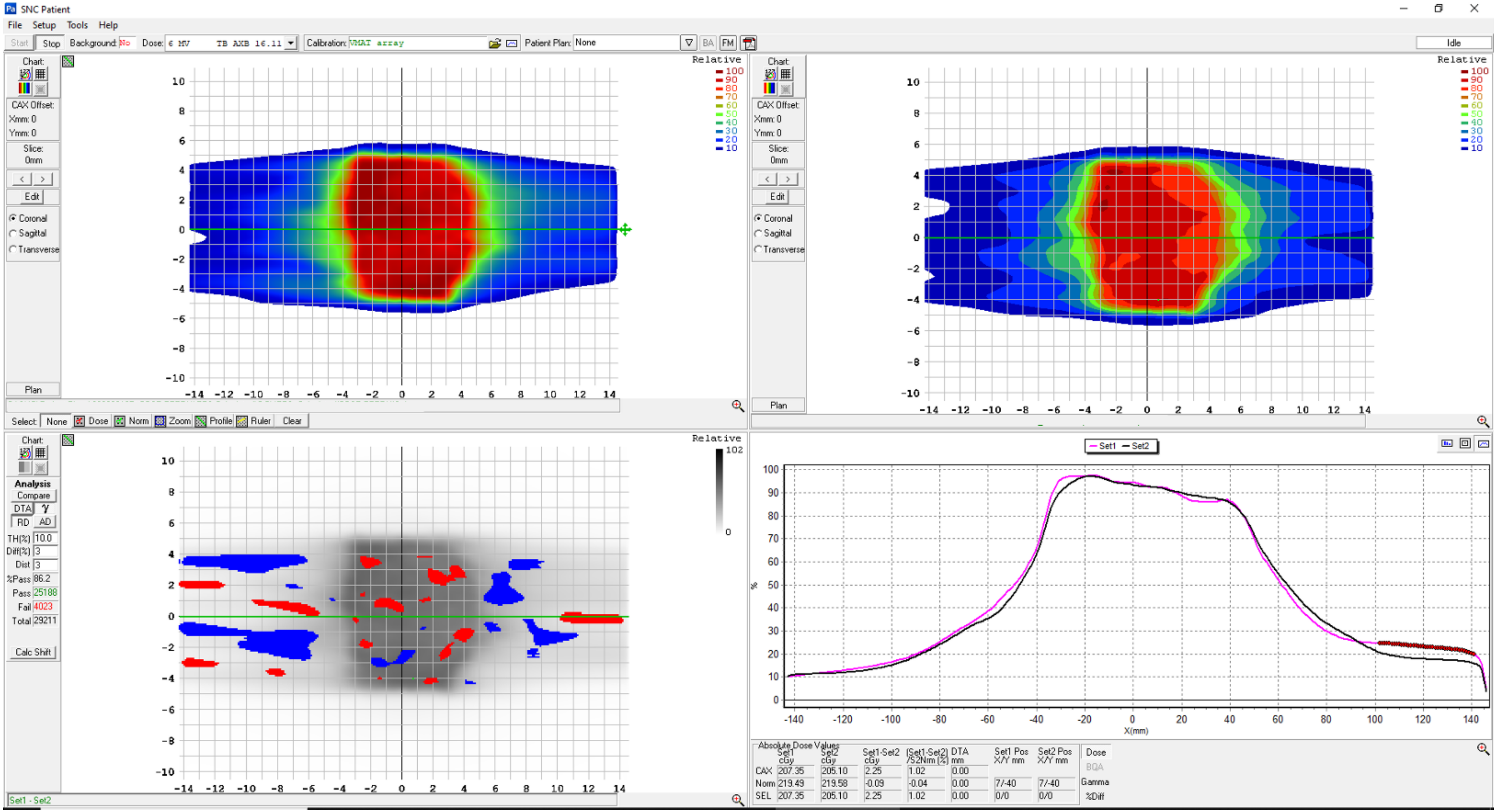

Figure S3. Gamma analysis between Plan_Ref (left) and Plan_AIedit (right)

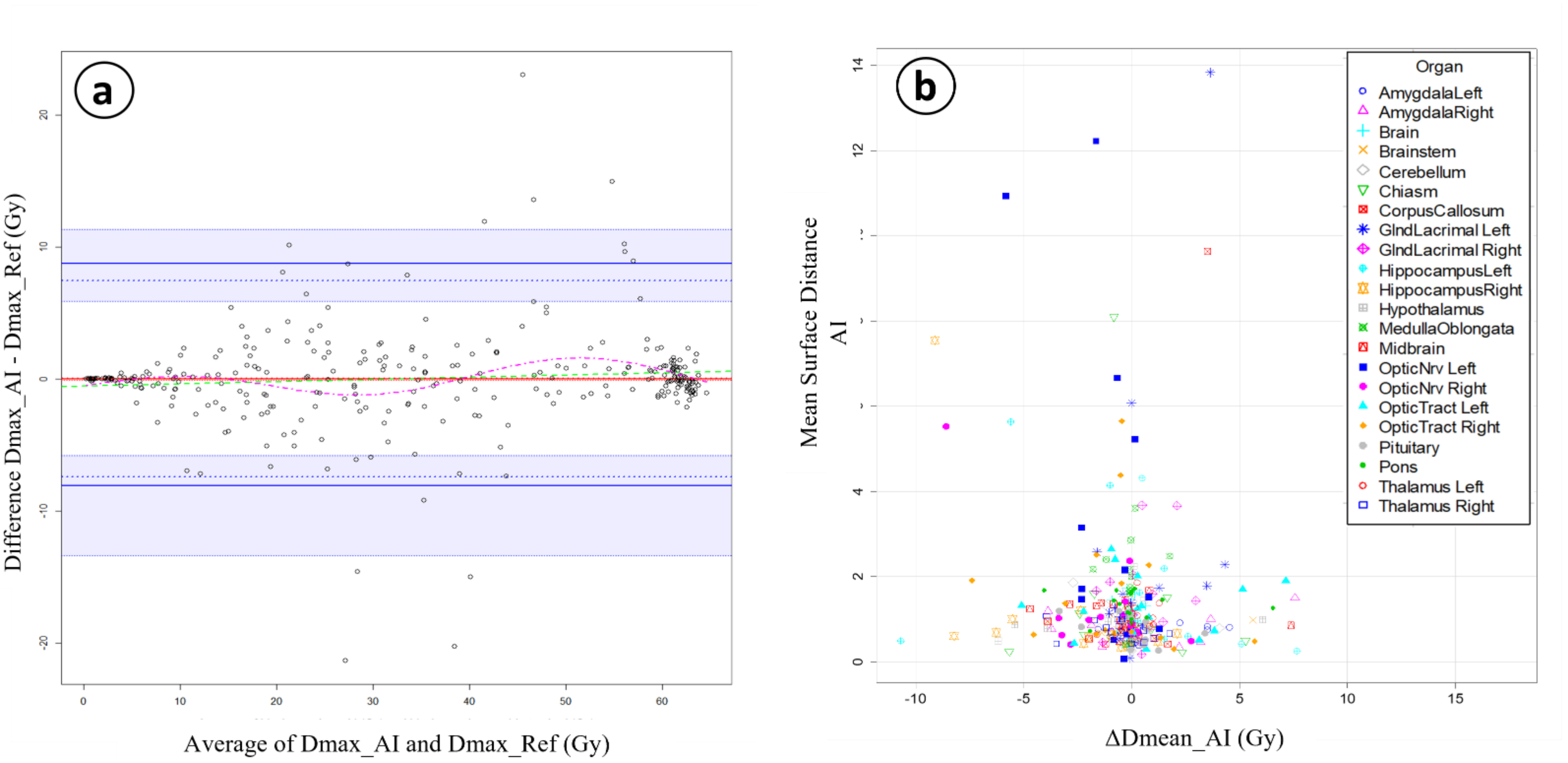

Figure S4. a: Bland-Altman plots showing the level of agreement between Plan_AI and Plan_Ref, in terms of and Dmax; **b:** Scatter plots showing the correlation between ΔDmean_AI (Gy) with the Mean Surface Distance (MSD_AI) for each OAR

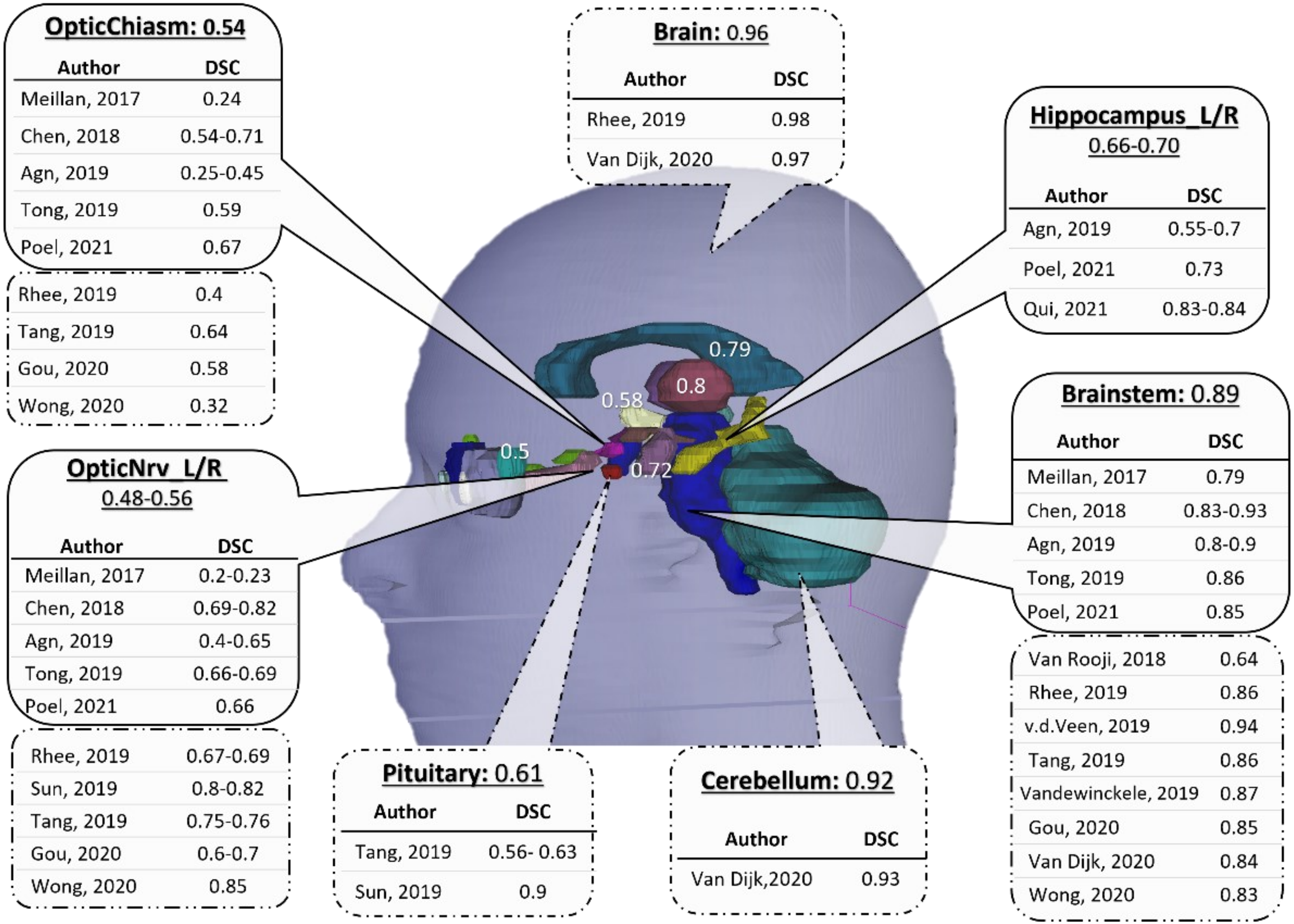

Figure S5. Overview of the published data regarding deep-learning AI-based auto-contouring performance in terms of DSC; Headings name the OAR and the DSC achieved in the current study; continuous lines encompass MRI-based studies and dashed lines CT-based reports. Numbers written near the OAR contours represent DSC_AI for OARs included in the current study, but for which there is no data for comparison.
